# Preparation of Electrospun Polyvinyl Alcohol/Nanocellulose Composite Film and Evaluation of Its Biomedical Performance

**DOI:** 10.3390/gels7040223

**Published:** 2021-11-19

**Authors:** Xinbin Ji, Jing Guo, Fucheng Guan, Yuanfa Liu, Qiang Yang, Xin Zhang, Yi Xu

**Affiliations:** School of Textile and Material Engineering, Dalian Polytechnic University, Dalian 116034, China; j19818902913@163.com (X.J.); Liuyf@dlpu.edu.cn (Y.L.); q_yang@outlook.com (Q.Y.); 15942675120@163.com (X.Z.); xuyi5672@163.com (Y.X.)

**Keywords:** polyvinyl alcohol, nanocellulose, wound dressing, nanofiber membranes, electrospinning

## Abstract

Using polyvinyl alcohol (PVA) and nanocellulose (NC) as raw materials, PVA/NC nanofiber membranes were prepared by electrospinning. The hydrogen bonding, crystalline properties and microscopic appearance of PVA/NC membranes with different NC contents were characterized. The mechanical properties, liquid absorption and cytotoxicity of the nanofiber membrane were evaluated. The results show that the free hydroxyl group of the PVA/NC nanofiber membranes have a maximum value of 9% at a mass fraction of 6% NC. The crystallinity of the PVA/NC nanofiber membranes and the average diameter of the nanofibers decreased and then increased as the NC content increased, with a minimum value of 38.23% and 272.03 nm, respectively, at 6% NC content. At this time, the contact angle was the smallest. The maximum strength of the PVA/NC nanofiber membranes is 75.8% higher than that of the PVA membrane at 2% NC content. With increasing NC content, the absorption of water, PBS sustained-release suspensions and artificial blood by PVA/NC nanofiber membranes increases. Cytotoxicity tests have shown that PVA/NC nanofiber membranes are non-toxic, have good cytocompatibility and are expected to be used in the field of medical dressings.

## 1. Introduction

As the largest organ of the body, the skin is the body’s first barrier of defense, which plays an important role in controlling the body’s temperature and protecting the many capillaries and nerves inside the body [1]. However, trauma to the body is inevitable in daily life. According to statistics from the US military, in the last decade, one-quarter of deaths were related to improper wound management. The appropriate use of dressings is an effective way to avoid wound infection and accelerate wound healing. Numerous requirements should be taken into account when designing and manufacturing the ideal dressing, such as adequate mechanical properties, non-toxicity, good hydrophilicity, excellent biocompatibility and ability to absorb wound exudate, etc. Therefore, research on highly absorbent, non-toxic and anti-inflammatory medical dressings has become a hot topic [2,3].

Polyvinyl alcohol (PVA) is a semi-crystalline, water-soluble polyhydroxy polymer that is widely used in the textile, paper and healthcare sectors due to its good film-forming, hydrophilic, biocompatible and biodegradable properties [4,5]. With a high specific surface area, high porosity and the structure and function of a biomimetic extracellular matrix, PVA nanofiber membranes have irreplaceable advantages in medical dressings, biomedical materials and tissue engineering. However, PVA nanofiber membranes have some drawback such as low strength and limited absorption of liquids. A great deal of research has been carried out to address these problems.

Lu et al., prepared PVA/EW membranes using 12% PVA solution compounded with egg white (EW) by electrostatic spinning and found the mechanical properties and crystallinity of the fibers were improved by using the co-blending cross-linking method [6]. The prepared PVA/EW nanofiber membranes had a uniform porous, smooth fiber structure and good biocompatibility, which could significantly promote cell proliferation, demonstrating the feasibility of PVA/EW composites to prepare fiber dressings for skin defect repair and regeneration. Zhang et al., prepared ultra-fine PVA/graphene oxide (GO) nanofiber scaffolds (PGNSs) by electrostatic spinning and found PGNSs containing 0.25% GO had good hydrophilicity and protein enrichment, which was suitable for the growth and adhesion of L929 fibroblasts [7]. Safaee et al., prepared schizophyllan/PVA nanofibers by electrostatic spinning and then cross-linked them with glutaraldehyde vapour [8]. The results showed that the schizophyllan/PVA nanofiber mats with a volume ratio of 20:80 promoted cell proliferation and migration, making them a suitable material for promoting wound healing. Zou et al., prepared PVA/chitosan (PVA/CS) nanofibers with antimicrobial effect [9], which can effectively promote skin wound healing. Wang et al., prepared an environmentally friendly PVA/CS nanofiber membrane by electrostatic spinning and showed that the average diameter of the fiber was positively correlated with the amount of PVA added [10].

In order to expand the application of PVA in the field of high-strength materials, PVA is often used as a polymer matrix and nanocellulose (NC) is used as a reinforcing agent in composite materials. NC and its nanocrystals (CNC) have the advantages of regular structure, high crystallinity and large specific surface area [11]. NC has polar hydroxyl groups that can form hydrogen bonds with polysaccharides; thus, it is touted as the most promising reinforcing materials of the 21st century [12,13]. Through the formation of intermolecular or intramolecular hydrogen bonds between PVA and the hydroxyl groups of NC, the purpose of enhancing the performance of PVA composites can be achieved. Zhang et al., prepared a water-washable PVA/CNC nanofiber air filter by electrostatic spinning for particulate matter removal and found that water resistance of air filter can be improved by a simple heat treatment without adding any crosslinker [14]. Elliott et al., prepared PVA/CNC composites by electrostatic spinning [15], and the mechanical properties of composites were reinforced by thermal treatment. Anna et al., prepared PVA/NC nanofibers by electrostatic spinning, which improved the crystallinity of the nanofiber membranes [16], leading to an increase in both stability and melting temperature. However, the previous study did not investigate the effects of hydrogen bonding interactions between PVA and NC on the various properties of composites. Furthermore, the biocompatibility of PVA/NC composite membranes has been rarely reported.

In this study, we optimized the spinning solution PVA/NC ratios by Zeta potential, and then prepared PVA/NC nanofiber membranes by electrostatic spinning. The PVA/NC nanofiber membranes at different concentrations were analyzed by quantitative infrared analysis methods and Gaussian fitting. The form of hydrogen bonding between PVA and NC and its mechanism were investigated. The crystalline properties, diameter size distribution, mechanical properties, hydrophilicity and liquid absorption properties were studied by hydrogen bonding content. The cytotoxicity of the nanofiber membranes was evaluated. The results showed that the PVA/NC nanofiber membranes prepared have high strength and high absorbency, are non-toxic and are expected to be used in the field of medical excipients.

## 2. Results and Discussion

### 2.1. ZETA Potential of the Spinning Solution

**Figure 1:**
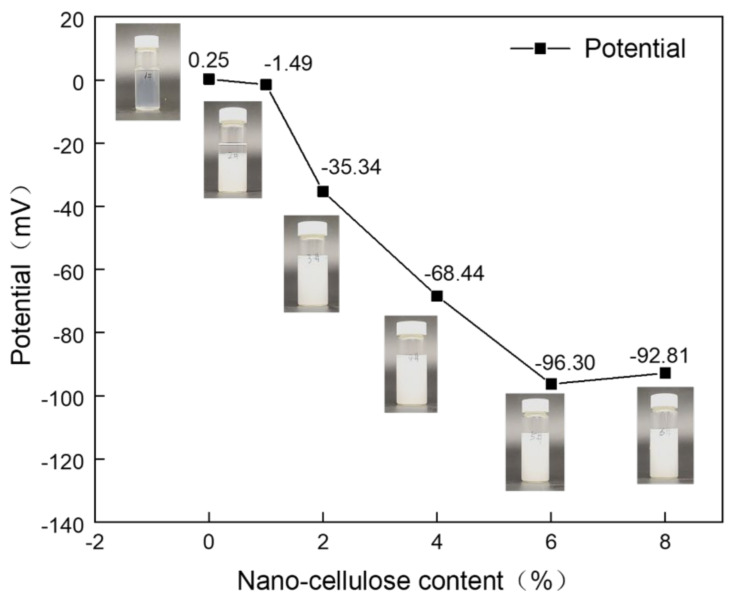
Zeta potential of spinning solutions with different NC mass ratios.

The solution is generally considered to be stable when the absolute value of the potential is greater than 30 mV [17,18]. As can be seen from Figure 1, the potential value of NC1 solution is −1.49 mV, which is less than 30 mV. It has been shown that the separation of the two phases occurs when left for five days, proving that the solution is unstable. The absolute potential values of the spinning solutions NC2–NC8 are all greater than 30 mV, indicating the solutions are stable. The spinning solutions NC0, NC2, NC4, NC6 and NC8 were selected for electrostatic spinning, while NC0 was used as a control.

### 2.2. The Structure and Hydrogen Bond Fitting of PVA/NC Nanofiber Membranes

**Figure 2:**
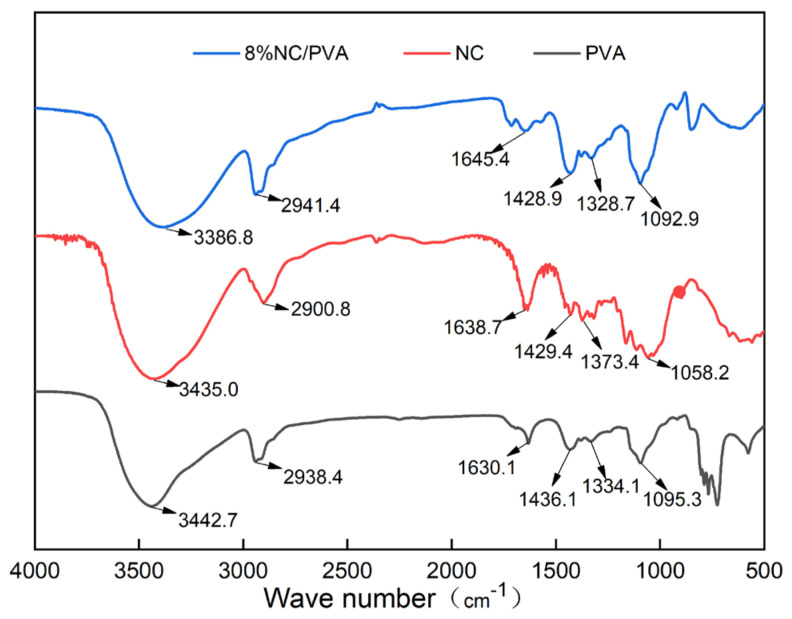
Infrared spectra of PVA, NC and PVA/NC composite membranes.

Both PVA and NC have strong hydroxyl peaks. As shown in Figure 2, the —OH stretching vibrations of PVA and NC are around 3443 cm^−1^ and 3435 cm^−1^, respectively, and after compounding the characteristic peak of —OH is shifted to around 3386 cm^−1^. For NC, the bending vibration absorption peak of CH_2_ appears at 1429 cm^−1^ and ring stretching vibration absorption peak appears at 1058 cm^−1^, respectively. The CH_2_ stretching vibration and bending vibration peaks in the PVA alkyl group appear near 2938 cm^−1^ and 1436 cm^−1^, respectively. For the PVA/NC composite membranes, they move to near 2941 cm^−1^ and 1428 cm^−1^, respectively. There is no new absorption peak in the composite material, which may be due to the strong hydrogen bonding interaction between the composite material molecules [14]. The form of intermolecular hydrogen bonding between NC and PVA has a strong influence, which weakens the interactions between similar molecules and enhances the combining performance between PVA and NC, ensures the high spinnability of PVA/NC.

**Figure 3:**
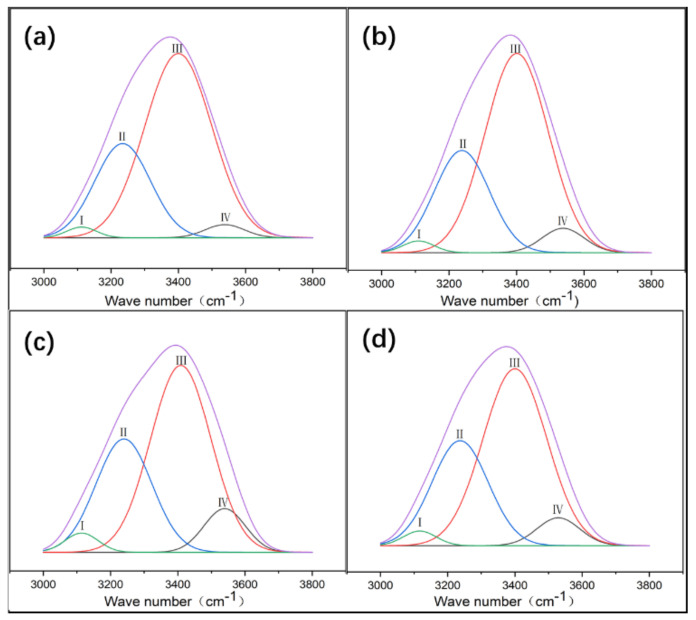
Gaussian curve fitting of different types of hydrogen bonds of PVA/NC nanofibers with different NC content to composite materials. (**a**) The proportion of different types of hydrogen bonds in NC2 nanofibers. (**b**) The proportion of different types of hydrogen bonds in NC4 nanofibers. (**c**) The proportion of different types of hydrogen bonds in NC6 nanofibers. (**d**) The proportion of different types of hydrogen bonds in NC8 nanofibers.

**Figure 4:**
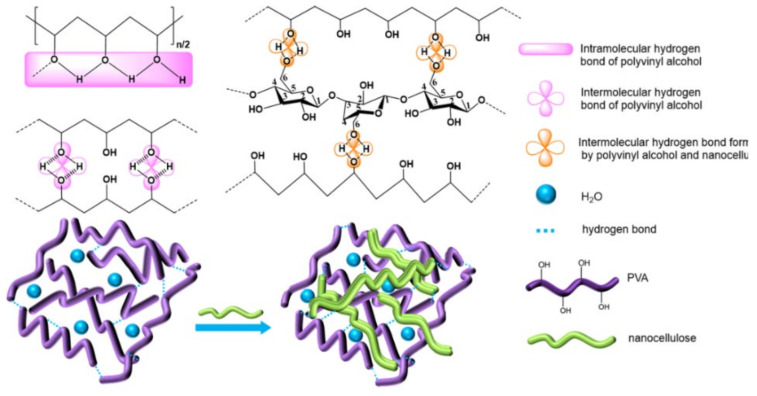
The hydrogen bonding interaction between PVA and NC.

There are many hydrogen bond donors and acceptors in NC and PVA, resulting in many different types of hydrogen bonds formed in the complex, such as OH…OH, OH…ether O, —OH annular polymer. According to the references, the attribution of different types of hydrogen bonds in infrared spectroscopy has been confirmed [19]. In order to determine the hydrogen bonding interaction, Gaussian peak fitting was performed on the 3000–3800 cm^−1^ peak in the FT-IR spectrum. In the second-order derivative spectrum, it is found that PVA/NC composite membranes have four peaks, and the proportion of each peak is shown in Figure 3. The characteristic absorption peak of the free hydroxyl group is around 3570 cm^−1^, the characteristic absorption peak of OH…OH is around 3400 cm^−1^ (including the hydrogen bonding interaction of —OH within the PVA molecule and the hydrogen bonding interaction of PVA with —OH of the NC molecule); the characteristic absorption peak of —OH annular polymer is around 3118 cm^−1^ (including the hydrogen bonding interaction of —OH within the PVA molecule), and the characteristic absorption peak of OH…ether O is around 3217 cm^−1^ [20,21] (including hydrogen bonding between —OH of PVA and —O— of NC). The hydrogen bond formation mechanism is shown in Figure 4. The area under the curve corresponding to each characteristic peak represents the composition ratio of various hydrogen bonds, and the results are shown in Table 1.

Table 1 shows that the proportion of intermolecular hydrogen bonds increases and then decreases as the NC content increases, reaching a maximum value of 31.43% at the NC content of 6%. The proportion of intramolecular hydrogen bonds decreases and then increases with the increase of NC content, reaching a minimum value (59.57%) at 6% NC content. As shown in Figure 4, due to a large number of hydroxyl and ether bonds on the surface of NC, NC will act as a hydrogen bond donor to replace the intramolecular and intermolecular hydrogen bonding of PVA, resulting in an increase in intermolecular hydrogen bonding and a decrease in intramolecular hydrogen bonding. At 8% NC content, the percentage of intermolecular hydrogen bonds decreases. This may be due to the agglomeration of NC and the reduction of bare hydroxyl and ether bonds, which ultimately leads to a reduction in the effective dispersion surface within the PVA matrix.

### 2.3. Crystallization Properties of PVA/NC Nanofiber Membranes

**Figure 5:**
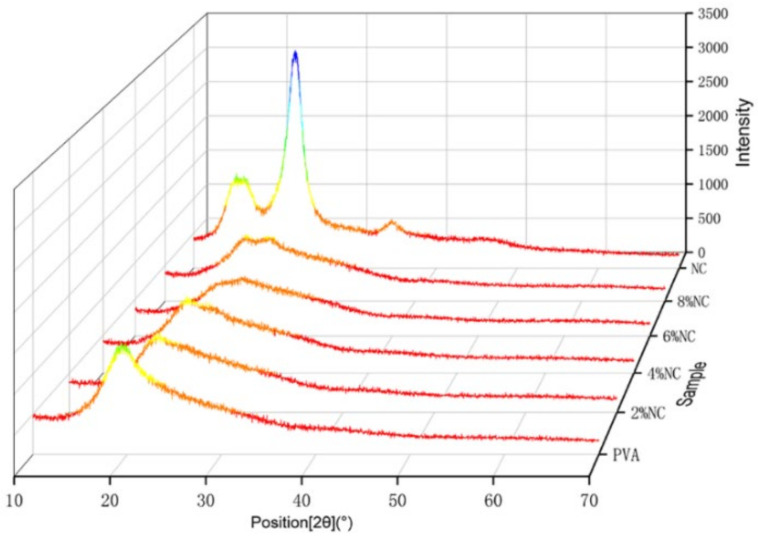
XRD patterns of PVA/NC nanofiber membranes with different NC content and pure NC.

It can be clearly seen from Figure 5 that PVA has clear diffraction peaks at 2θ = 19.4°, corresponding to [101] crystalline plane [22]. The clear diffraction peaks of NC near 2θ = 15.36°, 22.54° and 34.27°, which are typical of type I cellulose [002], [101] and [004] crystalline planes, respectively, indicating the typical crystalline characteristics of natural cellulose [23,24,25]. At 6% NC content, the PVA/NC nanofiber membranes has a weaker double peak at the position of the corresponding diffraction peaks of PVA and NC (2θ = 19.4° and 22.54°). At an NC content of 8%, the PVA/NC nanofiber membranes show a clear double peak. It indicates that the strong interaction between PVA and NC did not change the respective crystalline shapes, but weakened the respective crystallinity of PVA and NC, resulting in the decrease in crystallinity with increasing NC content, reaching a minimum value (38.23%) at 6% NC content (e.g., Table 2).

At 8% NC content, the crystallinity of the PVA/NC nanofiber membranes rebounds, probably because the intermolecular hydrogen bonding between NC and PVA is weakened, while the intramolecular hydrogen bonding is increased. Eventually the two interact to form aggregates into crystalline nuclei, increasing the nucleation sites and leading to an increased crystallinity. It is the decrease in crystallinity that lays the structural basis for the high liquid absorbency of the PVA/NC nanofiber membranes.

### 2.4. Morphology and Porosity of PVA/NC Nanofiber Membranes

**Figure 6:**
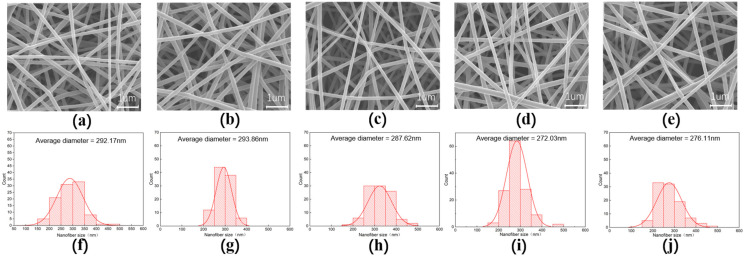
The SEM images and fiber diameter distribution diagrams of PVA/NC nanofiber membranes with different NC content. (**a**,**f**) are the electron microscope image and diameter distribution diagram of NC0, respectively. (**b**,**g**) are the electron microscope image and diameter distribution diagram of NC2, respectively. (**c**,**h**) are the electron microscope image and diameter distribution diagram of NC4, respectively. (**d**,**i**) are the electron microscope image and diameter distribution diagram of NC6, respectively. (**e**,**j**) are the electron microscope image and diameter distribution diagram of NC8, respectively.

From the morphology of the nanofiber membranes in Figure 6, we can see that the NC0–NC8 nanofibers have uniform fiber diameter, no bonding, no bead, smooth surfaces and good fiber morphology.

The NC6 nanofibers have the smallest average diameter with a highly concentrated diameter distribution. This is because as the content of NC in the solution increases, the total amount of intermolecular and intramolecular hydrogen bonds decreases (91%), and the free —OH increases (9%). The viscosity of the solution system decreases, the resistance to flow decreases and the fine flow of solution is more susceptible to tensile deformation in the electrostatic field, which in turn leads to a decrease in the nanofiber diameter [26]. However, at 8% NC content, the total amount of intramolecular and intermolecular hydrogen bonds increased (93.65%), the free —OH decreased (6.35%), the viscosity of the solution system increased, the resistance to deformation increased and the nanofiber diameter increased. This indicates that the free —OH content can influence the PVA/NC nanofiber diameter to some extent.

**Figure 7:**
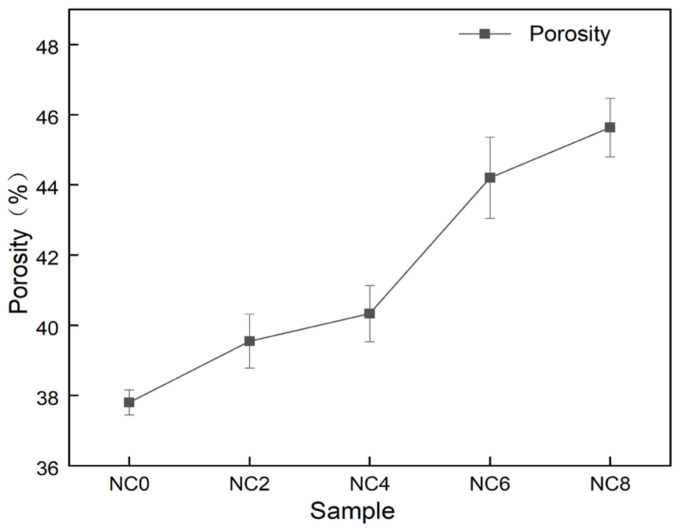
Porosity of PVA/NC nanofiber membranes with different content of NC.

The nanofiber membrane produced by electrospinning technology has a large specific surface area [27,28], and the nanofibers overlap each other to form a pore structure. The porosity of nanofiber membranes with different mass ratios was calculated by fomula (1). It can be seen from Figure 7 that the porosity of PVA nanofiber membranes was 38.20%, and the porosity of PVA/NC nanofiber membranes monotonically increased with the increase of NC content. The porosity of NC8 nanofiber membranes increased to 45.94%, an increase of 120% compared to PVA. This is because as the NC content increases to 6%, the PVA/NC nanofiber diameter decreases and the nanofiber membrane density gradually increases, leading to an increase in the porosity of the PVA/NC nanofiber membrane [29]. However, at 8% NC content, the increased rigidity of the NC reduces the potential adhesion points between the PVA/NC nanofibers, allowing for an increase in the voids between the nanofibers, ultimately leading to an increase in porosity. The PVA/NC nanofiber membranes have a higher porosity than the common polymeric membranes (30–40%), which facilitates the absorption of body fluids [30].

### 2.5. Mechanical Properties of PVA/NC Nanofiber Membranes

**Figure 8:**
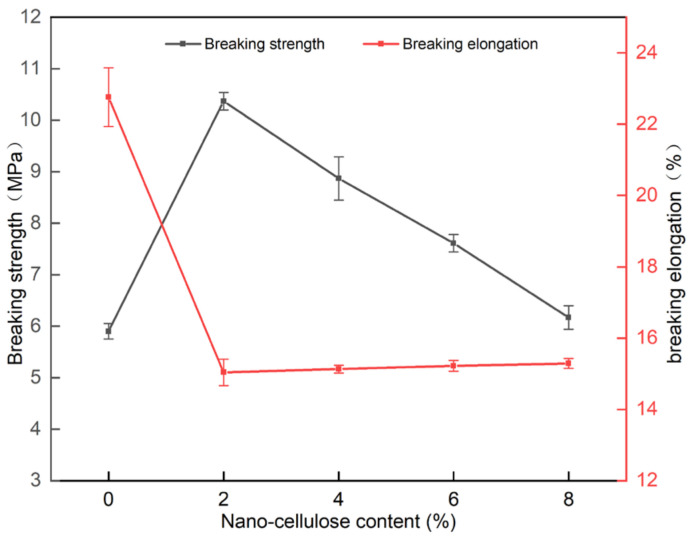
Breaking strength and breaking elongation of PVA/NC nanofiber membranes with different content of NC.

As shown in Figure 8, the fracture strength of the PVA/NC nanofiber membranes was generally greater than that of PVA, reaching the best mechanical properties of 10.37 MPa at 2% NC content, an increase of 75.8% compared to pure PVA membrane. The elongation at break of the PVA/NC nanofiber membranes decreased compared with PVA membrane, but it was at a level of 15%. This is because the NC2 nanofiber membranes, compared to the pure PVA membrane, introduce rigid NC, which increases intermolecular interactions and forms high-density hydrogen bonding clusters in the spatially restricted domain. NC can be uniformly present in the composite nanofiber membranes to play its role as a reinforcing body, which can play a stress-sharing role when subjected to external forces, making the fracture strength greatly improved and the toughness decreased. As the NC content increases, the porosity increases and the nanofiber diameter decreases, resulting in a decrease in fracture strength and an increase in toughness.

### 2.6. Contact Angle of PVA/NC Nanofiber Membranes

**Figure 9:**
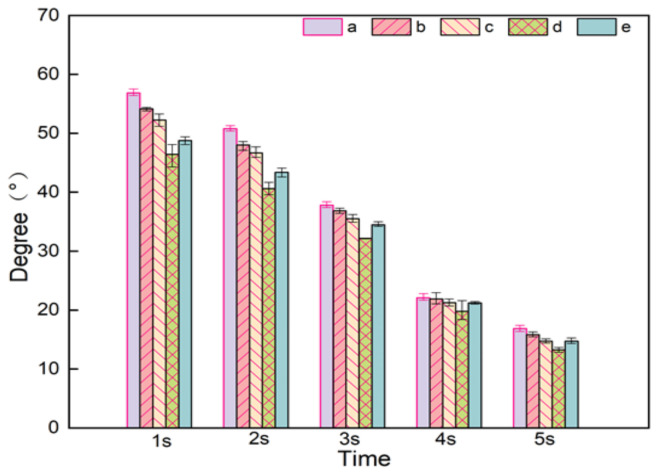
Contact angles of (**a**) NC0, (**b**) NC2, (**c**) NC4, (**d**) NC6 and (**e**) NC8 nanofiber membranes at different times.

Both PVA and NC in composite nanofiber membranes contain a large number of hydroxyl groups, both of them can form strong hydrogen bonding forces with water [31]. It can be seen from Figure 9, the contact angle of PVA/NC nanofiber membranes decreases with increasing NC content, indicating that the addition of NC can improve its water resistance to a certain extent. The hydrophilicity is related to the crystallinity of PVA/NC nanofiber membranes. At 6% NC content, the contact angle is the smallest. This is because the crystallinity of the PVA/NC nanofiber membranes reaches the lowest level, the proportion of amorphous regions reaches the highest level and more —OH binds to the water molecules, thereby increasing the hydrophilicity.

### 2.7. Liquid Absorption Performance of PVA/NC Nanofiber Membranes

**Figure 10:**
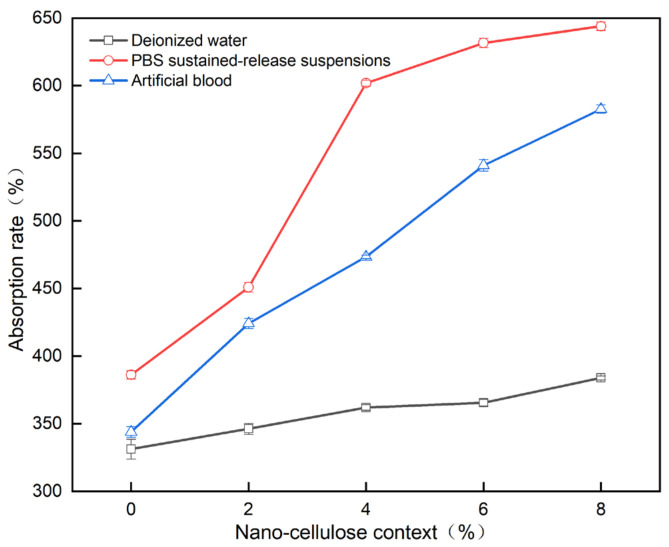
Liquid absorption rate of PVA/NC nanofiber membranes with different content of NC.

**Figure 11:**
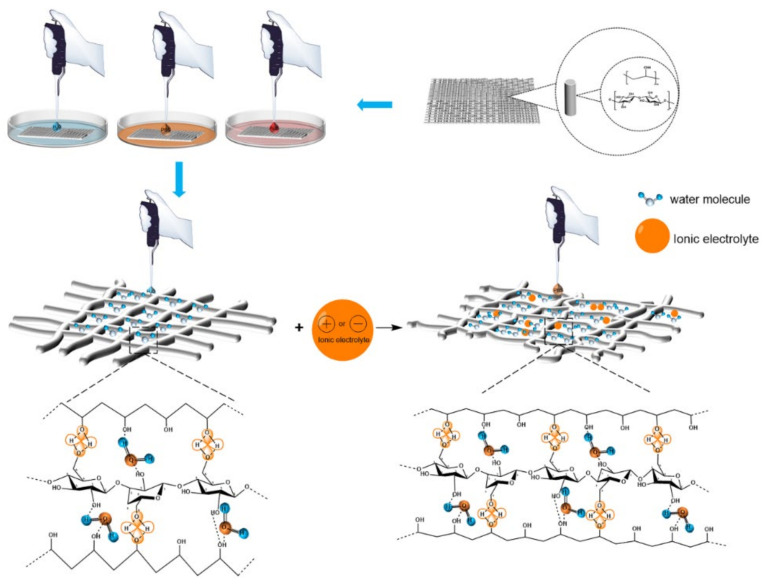
The interaction mechanism between PVA/NC nanofiber membranes with different content of NC and liquids.

As shown in Figure 10, the absorption of water, PBS sustained-release suspensions and artificial blood by the PVA/NC nanofiber membranes increased as the NC content increased. For the same NC mass fraction, the absorption of PBS by the PVA/NC nanofiber membranes was greater than that of artificial blood and water. This is partly due to the fact that the diameter of the nanofibers decreases as the NC content increases, and the specific surface area and porosity of the nanofiber membranes increase, ultimately leading to an increase in the amount of liquid absorption. On the other hand, PBS, artificial blood and water are small molecules, the absorption of which by nanofibrous membranes occurs self-energetically in the amorphous zone and at the edges of the crystalline zone. The addition of NC reduces the crystallinity and increases the amorphous zone, making the small molecules more accessible. The difference in the absorption performance of nanofiber membranes for PBS, artificial blood and water are related to the interaction mechanism of the nanofiber membranes with the three liquids in Figure 11.

As shown in Figure 11, there are numerous electrolytes in PBS and artificial blood, with the addition of electrolytes, the effective size of the molecules shrinks and the free volume increases, resulting in larger voids and permeable areas being created. The number of hydrogen bonds generated in the region increases and the crystalline regions bound to water are partially converted into amorphous regions, leading to a decrease in crystallinity and an increase in the accessibility of the absorbed liquid. In the case of artificial blood, which contains 6% hydroxyethyl starch, the viscosity is greater than that of PBS, which slows down the movement of small molecules and results in a smaller absorption rate than PBS.

### 2.8. Cytotoxicity of PVA/NC Nanofiber Membranes

**Figure 12:**
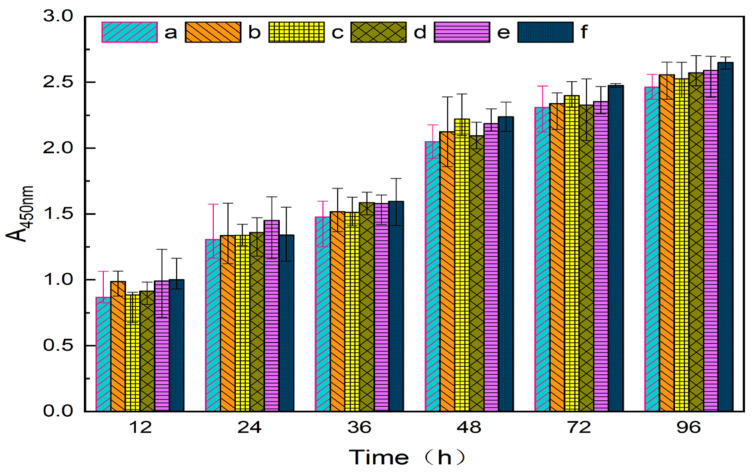
Cytotoxicity of nanofiber membranes in (**a**) blank group and (**b**) NC0, (**c**) NC2, (**d**) NC4, (**e**) NC6 and (**f**) NC8.

As shown in Figure 12, the cell proliferation rates of the extracts of different contents of PVA/NC nanofiber membranes were all greater than 100%, indicating that they were not cytotoxic. In addition, the cell value-added rates of PVA/NC nanofiber membranes were all higher than those of pure PVA nanofiber membranes, indicating that NC does not affect cell growth and differentiation [32]. It indicated that PVA/NC nanofiber membranes are biocompatible and can be expected to be used as medical dressings.

## 3. Conclusions

PVA/NC nanofiber membranes were prepared by electrostatic spinning with fiber diameters ranging from 150 to 450 nm, and the smallest average fiber diameter of 272.03 nm at an NC mass fraction of 6%. Infrared spectroscopy and its Gaussian fit analysis show that there is a minimum value of intramolecular and intermolecular hydrogen bonding summation with increasing NC content in PVA/NC nanofiber membranes. Intramolecular hydrogen bonding is dominated by OH…OH, and intermolecular hydrogen bonding is dominated by OH… ether O. The maximum free hydroxyl content was 9% at 6% NC, when the minimum crystallinity of the PVA/NC membranes was 38.23%. With the addition of NC, the PVA/NC membranes showed a 75.8% increase in fracture strength, a reduction in contact angle and improved hydrophilicity compared to PVA membranes. The absorption of PVA/NC nanofiber membranes for deionized water, PBS sustained-release suspensions and artificial blood increases with the increase of NC content. The maximum absorption of the three liquids was 380%, 650% and 580%, respectively. The results show that the PVA/NC nanofiber membranes with 6% NC content have the smallest fiber diameter and are more consistent with the structure and function of the extracellular matrix. The PVA/NC nanofiber membranes are expected to be used in the field of wound dressings due to its non-toxic, better hydrophilic properties, higher mechanical strength and water absorption.

## 4. Experiments and Methods

### 4.1. Materials

Polyvinyl alcohol (PVA, type 1799, alcoholysis degree: 98–99%, polymerization degree 1750 ± 50) was purchased from Aladdin Chemical Reagent Co., Ltd. (Shanghai, China). Nanocellulose (length 119.7 nm, diameter 3.31 nm) was purchased from Dalian Institute of Chemical Physics, Chinese Academy of Sciences. The RSC96 cell line was purchased from Guangzhou Jinio Biotechnology Co., Ltd. (Guangzhou, China). The CCK-8 Kit (Cell Counting Kit-8) was purchased from Biosharp White Shark Biotechnology Co., Ltd. (Shanghai, China). Cell culture medium (sterile filtration) was purchased from Ge Healthcare Life Sciences Hyclone Co., Ltd. (Beijing, China). Medical alcohol (70–75%) was purchased from Panjin Tianyuan Pharmaceutical Co., Ltd. (Liaoning, China). Phosphate-buffered saline (PBS, powder) was purchased from Beijing Soleibao Technology Co., Ltd. (Beijing, China). Artificial blood was purchased from Dongguan Chuangfeng Technology Co., Ltd. (Guangdong, China).

### 4.2. Preparation of PVA/NC Nanofiber Membranes

**Figure 13:**
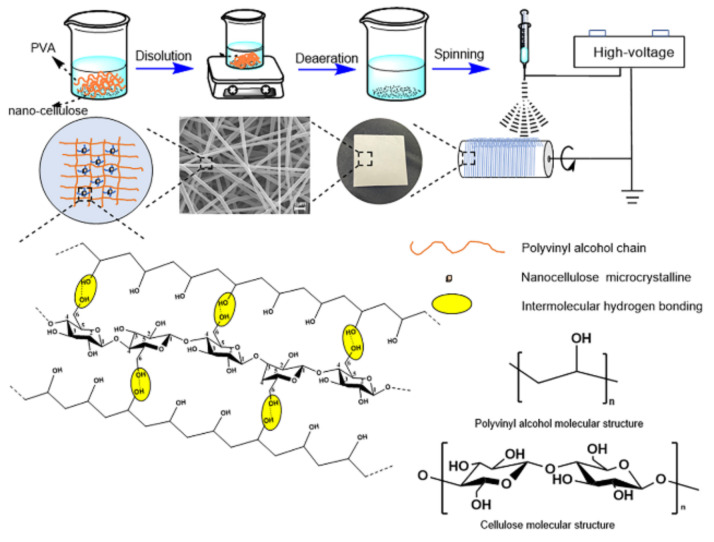
Preparation scheme of PVA/NC nanofiber membranes.

A certain amount of PVA and deionized water was taken and stirred in an oil bath at 500 rpm and 97 °C for 2 h. Then, different contents of nanocellulose emulsion (NC) were added and stirred at room temperature for 12 h to obtain PVA/NC spinning solution, where the concentration of PVA was 8%. The PVA/NC nanofiber membrane preparation scheme is shown in Figure 13. The configured PVA and PVA/NC spinning solution was left to defoam, and then the PVA and PVA/NC spinning solutions were placed in a 20 mL syringe (21 G) and spun using a TL-Pro-BM high-pressure electrostatic spinning machine manufactured by Shenzhen Tongli Micro-Nano Technology Co. Spinning conditions: voltage 22.00 ± 1 kV, temperature 34 ± 1 °C, humidity 10 ± 2%, flow rate 1 mL/h.

### 4.3. ZETA Potential Test of Spinning Solution

The NC content was 0 wt%, 1 wt%, 2 wt%, 4 wt%, 6 wt% and 8 wt%, respectively, noted as NC0, NC1, NC2, NC4, NC6 and NC8, using Delsa Nano C (Japan Beckman Coulter) performed potential measurements on different samples.

### 4.4. PVA/NC Nanofiber Membranes Structure and Performance Characterization

#### 4.4.1. Chemical Structure of PVA/NC Nanofiber Membranes

The chemical structure of the PVA/NC nanofiber membranes was analyzed using a Spectrum-One B infrared spectrometer (FT-IR, PE Spectrum two, Norwalk, Connecticut, USA). The PVA/NC nanofiber membranes were crushed, and the sample were prepared by KBr compressions and tested in the wave number range of 4000 to 400 cm^−1^.

#### 4.4.2. Crystallization Performance of PVA/NC Nanofiber Membranes

The crystalline properties of PVA/NC nanofiber membranes were tested using a Max-3B X-ray diffractometer (XRD, RIKEN, Matsubara Town, Akishima City, Tokyo, Japan). The test conditions are tube voltage 40 kV, tube current 2–80 mA, 2θ range 5°–70°, scanning speed 4 (°)/min. The test conditions are tube voltage 40 kV, tube current 2–80 mA, 2θ range 5°–70°, scanning speed 4 (°)/min.

#### 4.4.3. Microscopic Morphology, Diameter and Porosity of PVA/NC Nanofiber Membranes

The PVA/NC nanofiber membranes were observed using an S-4800 scanning electron microscope (SEM, HITACHI, Akishima City, Tokyo, Japan) with an accelerating voltage of 10 kV. The diameter distribution and average diameter of 100 nanofibers were calculated on the SEM image of the sample using Nano Measure software.

The electrostatically spun membranes were immersed in anhydrous ethanol for 24 h. The excess anhydrous ethanol was removed from the surface with absorbent paper, weighed and recorded as *W*_1_. Subsequently, the nanofiber membranes were dried in a vacuum drying oven at 80 °C for 24 h. The samples were then weighed and recorded as *W*_2_, and the porosity of the nanofibers was calculated according to the following Equation [33]:

(1) $$\mathrm{Pr}=\frac{W_{1}-W_{2}}{\rho AL_{d}}\times 100\%$$

Among them, Pr: porosity, ρ: the density of absolute ethanol (g/cm^3^), A: the surface area of the sample (cm^2^) and *L_d_*: the thickness of the sample (cm).

#### 4.4.4. Mechanical Test of PVA/NC Nanofiber Membranes

The nanofiber membrane was cut into rectangular strips with a specification of 0.5 cm × 4 cm, and its mechanical properties were tested by the YG004 single-fiber strength machine. The stretching speed is 15 mm/min. The calculation method of tensile strength σ (MPa) and elongation at break ε (%) is as follows:

(2) $$\sigma =\frac{F}{M\delta }$$

(3) $$\varepsilon =\frac{L}{L_{0}}\times 100\%$$

σ: breaking strength (MPa), *F*: breaking force (N), *M*: sample width (mm), *δ*: sample thickness (mm), *ε*: breaking elongation (%), *L0*: initial length of the sample (mm), *L*: Break length (mm).

#### 4.4.5. Contact Angle Test of PVA/NC Nanofiber Membranes

The contact angle of the PVA/NC nanofiber membranes was tested using a DSA25 contact angle measuring instrument (Kruss Scientific Instruments, Hamburg, Germany). A 10 μL deionized water drop was used to measure the contact angle. When the droplet falls on the surface of the nanofiber membrane, the contact angle value is calculated by software, and the image is taken at 1–5 s using a high-speed camera. Each sample is tested 5 times and the average value is taken.

#### 4.4.6. Liquid Absorption Test of PVA/NC Nanofiber Membranes

Nanofiber membranes of different mass ratios were vacuum-dried and then three equal volumes were taken for water absorption, absorption of PBS slow-release solution and absorption of artificial blood, respectively. The samples were cut to size and the initial dry mass (*m*_0_) was recorded; they were then submerged in deionized water, simulated PBS buffer (pH = 7.2) and artificial plasma, removed and the surface of the sample was blotted with filter paper and the wet mass (*m_w_*) was recorded. The absorption capacity of the nanofiber membranes is calculated using the following equation:

(4) $$W=\frac{m_{w}-m_{0}}{m_{0}}\times 100\%$$

#### 4.4.7. Cytotoxicity Test of PVA/NC Nanofiber Membranes

Herein, RSC96 Schwann cells were used to evaluate the cytocompatibility. The RSC96 cells were grown on a VD-850 tabletop clean bench (Guangzhou Genio Biotechnology, Guangzhou, China), placed in a BB-150 CO_2_ incubator at 37°C and 5% CO_2_ concentration for three days (Thermo Fisher Scientific Instruments, Waltham, MA, USA), the RSC96 cells were diluted and transplanted on a 96-well cell culture plate and the cell culture medium was aspirated out with a pipette after 24 h. The plates were removed at different times, the cell culture medium was aspirated out and an equal amount of CCK-8 polyculture medium mixture was added, and three chromatic aberration groups were set up in one corner of the plates. After 30 min, the absorbance of each well was measured at 450 nm with an enzyme marker and compared with the set blank group to determine the cytotoxicity by the cell value-added rate.

## Figures and Tables

**Table 1:** The fitting results of various kinds of hydrogen bonds.

Sample	Hydrogen Bond Type		Abbreviations	Wave Number/cm^−1^	Average Peak Area	Relative Strength/%	Relative Strength/%
NC2	Free hydroxyl	Ⅳ	—OH	3570	6.32	2.82	2.82
	Intramolecular hydrogen bond	Ⅲ	OH…OH	3400	150.53	67.11	68.84
		Ⅰ	Annular polymer	3118	3.9	1.73	
	Intermolecular hydrogen bond	Ⅱ	OH…ether O	3217	63.56	28.34	28.34
NC4	Free hydroxyl	Ⅳ	—OH	3570	10.91	5.23	5.23
	Intramolecular hydrogen bond	Ⅲ	OH…OH	3400	134.23	64.33	66.18
		Ⅰ	Annular polymer	3118	3.87	1.85	
	Intermolecular hydrogen bond	Ⅱ	OH…ether O	3217	59.65	28.59	28.59
NC6	Free hydroxyl	Ⅳ	—OH	3570	18.1	9	9
	Intramolecular hydrogen bond	Ⅲ	OH…OH	3400	113.43	56.4	59.57
		Ⅰ	Annular polymer	3118	6.37	3.17	
	Intermolecular hydrogen bond	Ⅱ	OH…ether O	3217	63.21	31.43	31.43
NC8	Free hydroxyl	Ⅳ	—OH	3570	17.07	6.35	6.35
	Intramolecular hydrogen bond	Ⅲ	OH…OH	3400	161.11	59.91	62.44
		Ⅰ	Annular polymer	3118	6.8	2.53	
	Intermolecular hydrogen bond	Ⅱ	OH…ether O	3217	83.94	31.21	31.21

**Table 2:** Fitting results of crystallinity of various nanofiber membranes.

NC0	NC2	NC4	NC6	NC8
50.06%	48.08%	43.32%	38.23%	42.45%

## Data Availability

The data presented in this study are available on request from the corresponding author.
